# A neural geometry approach comprehensively explains apparently conflicting models of visual perceptual learning

**DOI:** 10.1038/s41562-025-02149-x

**Published:** 2025-03-31

**Authors:** Yu-Ang Cheng, Mehdi Sanayei, Xing Chen, Ke Jia, Sheng Li, Fang Fang, Takeo Watanabe, Alexander Thiele, Ru-Yuan Zhang

**Affiliations:** 1 Brain Health Institute, National Center for Mental Disorders, Shanghai Mental Health Center, Shanghai Jiao Tong University School of Medicine and School of Psychology, Shanghai, People’s Republic of China; 2 Department of Cognitive, Linguistic and Psychological Sciences, Brown University, Providence, RI USA; 3 Biosciences Institute, Newcastle University, Framlington Place, Newcastle upon Tyne, UK; 4 School of Cognitive Sciences, Institute for Research in Fundamental Sciences, Tehran, Iran; 5 Department of Ophthalmology, University of Pittsburgh, Pittsburgh, PA USA; 6 Affiliated Mental Health Center and Hangzhou Seventh People’s Hospital, Zhejiang University School of Medicine, Hangzhou, People’s Republic of China; 7 Liangzhu Laboratory, MOE Frontier Science Center for Brain Science and Brain-machine Integration, State Key Laboratory of Brain-Machine Intelligence, Zhejiang University, Hangzhou, People’s Republic of China; 8 NHC and CAMS Key Laboratory of Medical Neurobiology, Zhejiang University, Hangzhou, People’s Republic of China; 9 School of Psychological and Cognitive Sciences and Beijing Key Laboratory of Behavior and Mental Health, Peking University, Beijing, People’s Republic of China; 10 IDG/McGovern Institute for Brain Research, Peking University, Beijing, People’s Republic of China; 11 Key Laboratory of Machine Perception (Ministry of Education), Peking University, Beijing, People’s Republic of China; 12 Peking-Tsinghua Center for Life Sciences, Peking University, Beijing, People’s Republic of China

**Keywords:** Human behaviour, Perception

## Abstract

Visual perceptual learning (VPL), defined as long-term improvement in a visual task, is considered a crucial tool for elucidating underlying visual and brain plasticity. Previous studies have proposed several neural models of VPL, including changes in neural tuning or in noise correlations. Here, to adjudicate different models, we propose that all neural changes at single units can be conceptualized as geometric transformations of population response manifolds in a high-dimensional neural space. Following this neural geometry approach, we identified neural manifold shrinkage due to reduced trial-by-trial population response variability, rather than tuning or correlation changes, as the primary mechanism of VPL. Furthermore, manifold shrinkage successfully explains VPL effects across artificial neural responses in deep neural networks, multivariate blood-oxygenation-level-dependent signals in humans and multiunit activities in monkeys. These converging results suggest that our neural geometry approach comprehensively explains a wide range of empirical results and reconciles previously conflicting models of VPL.

## Abstract (web summary)

Previous studies have proposed conflicting models of visual perceptual learning. Leveraging deep neural network modelling, human functional MRI imaging and multiunit recordings in macaques, Cheng et al. introduce a neural geometry approach to reconcile past findings. They propose a unified theory of visual perceptual learning.

## Main

Adapting to new visual environments is crucial for an organism’s survival in its environment. This ability is well exemplified by visual perceptual learning (VPL), which is defined as long-term performance enhancements resulting from visual experience^1,2^. However, despite years of research in systems neuroscience, psychophysics and machine learning, the mechanisms behind VPL remain mysterious.

**Fig. 1:**
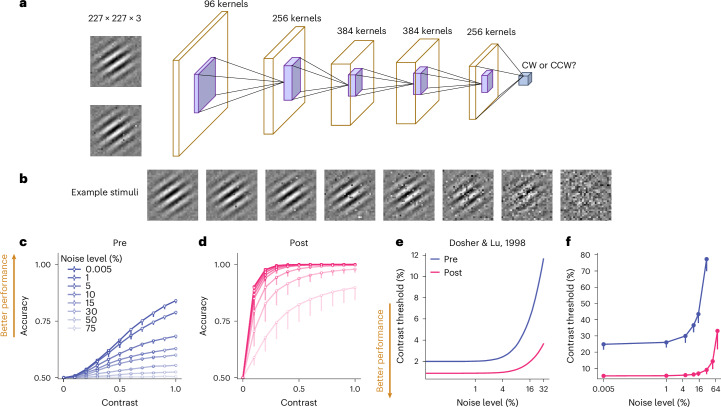
DCNN Modelling of orientation VPL. **a**,**b**, A DCNN (**a**) is trained on an orientation discrimination task (clockwise, CW or counterclockwise, CCW) with Gabor stimuli embedded in different levels of image noise (**b**). **c**,**d**, Orientation discrimination accuracy is improved from pre-test (**c**) to post-test (**d**). **e**,**f**, Training induces a downshift of the threshold versus noise function (**f**), an effect that is qualitatively similar to existing human psychophysical results (**e**, corresponds to the 70.7% accuracy condition in fig. 1 of ref. ^7^). The absolute quantitative differences between **e** and **f** may be due to differences in the overall SNR or the number of layers and units between the human visual system and the DCNN. Data are presented as mean ± s.e.m, with error bars and error shadings in **c** and **d** representing the s.e.m. across four (*n* = 4) reference orientations.

It is widely acknowledged that visual training enhances behavioural performance and refines representations in neural populations. Previous studies using human neuroimaging and monkey neurophysiology have demonstrated a significant improvement in the fidelity of stimulus encoding within population responses^3–5^. These findings strongly support the theory that enhanced signal-to-noise ratios (SNRs) serve as a potent computational mechanism for improved neural representations associated with VPL (Fig. 1e)^6–8^. However, improved SNR is an algorithm-level model, and the exact underlying neural mechanisms to achieve improved SNR remain elusive. Several conflicting models have been proposed on the basis of neural changes associated with VPL. One model suggests that VPL is associated with changes in population representations resulting from changes in neuronal tuning curves, as indicated by sharpened orientation tuning curves in monkey visual cortex^9,10^. Another model assumes that changes in population representations result from a reduction in trial-by-trial co-variation of neuronal firing rate, known as noise correlations, which have been observed in association with VPL in both monkeys and songbirds^11–14^.

The primary conceptual gap in reconciling the conflicting models of VPL lies in their focus on mechanisms proposed at the single-unit level (for example, changes in tuning curves or noise correlations), whereas the effects of these mechanisms in VPL should be evaluated at the population level (that is, improved population representations). Although VPL is indeed associated with changes in both single-unit responses and improved population representations, it remains controversial whether changes in single-unit responses are the actual cause or merely by-products of improved population representations. While these conflicting models of VPL capture certain aspects of the empirical findings, they fail to generate falsifiable predictions about how changes in single-unit responses contribute to improved population representations.

A major obstacle to comparing the conflicting models of VPL is the complex interactions between different aspects of single-unit responses (for example, tuning curves and noise correlations) on population representations. Computational neuroscience research has elucidated that the impact of noise correlations on population representations heavily depends on its interaction with tuning curves^15^. It is important to note that reduced noise correlations do not inherently enhance information in a neural population^16–18^. Moreover, the challenge is exacerbated by the fact that their interaction effects are even changing rather than remaining stable throughout a training process. These dynamic changes further complicate the understanding of how training affects their interactions. To overcome this, a comprehensive computational approach is imperative to quantify and disentangle the effects of different changes in single-unit responses, such as sharpened tuning curves and reduced noise correlations, on neural representations at the population level.

To comprehensively explain these conflicting models, we developed a neural geometry approach of VPL. In this approach, trial-by-trial population responses elicited by two stimuli for discrimination form two differentiable manifolds in a high-dimensional neural space. In this space, changes in single-unit responses (for example, tuning curves, Fano factor and noise correlations) can be interpreted as changes in several fundamental and measurable geometric properties (for example, centroids, size and orientations) of neural manifolds. This approach allows quantitative comparisons of conflicting models of VPL and assessments of their contributions to population representations within the same computational framework. Thus, this approach directly bridges single-unit responses and population representations and offers a normative account of the potential neural mechanisms underlying VPL. Specifically, this approach proposes four possible training-induced geometric changes (signal enhancement, manifold shrinkage, signal rotation and manifold warping) that can summarize all previous models of VPL. Thus, improved population representations can be achieved by one or a combination of the four interpretable mechanisms.

Our study includes theoretical modelling and empirical tests of model predictions. First, to assess this neural geometry approach, we trained deep convolutional neural networks (DCNNs) on the typical VPL task—orientation discrimination learning—and found that the DCNNs successfully replicated a wide range of psychophysical and imaging findings in humans, as well as neurophysiological findings in monkeys. Second, analyses of the geometric mechanisms mentioned above suggest that changes in both tuning curves and noise correlations are indeed present in VPL. Third, and most importantly, our analysis further revealed that neither changes in tuning curves nor changes in noise correlations at the single-unit level contributed significantly to improved population representations. Surprisingly, we found that neural manifold shrinkage induced by reduced response variability emerged as the primary mechanism driving VPL. Our neural geometry approach generates several empirical testable predictions. We directly tested these predictions on empirical data across different tasks, different levels of measurement and different brain regions in different species. Remarkably, we found that the geometry approach incorporating manifold shrinkage aligned closely with the activity of artificial neurons in DCNNs trained on VPL of motion direction discrimination learning task, blood-oxygenation-level-dependent (BOLD) response changes associated with VPL of motion direction learning in humans, and the electrophysiological population response changes associated with VPL of contrast discrimination in monkey V4.

## Results

### VPL improves behavioural performance of DCNN

To elucidate the neurocomputational mechanisms of VPL, we trained a DCNN (Fig. 1a) to perform a classical orientation discrimination task^7^. DCNN modelling allows us to easily assess the activity of the whole population in each layer and along the entire visual hierarchy. Similar to the neural network in ref. ^19^, this neural network inherits the first five convolutional layers of AlexNet, which was pretrained on ImageNet^20^. To emulate the decision stage of orientation discrimination, we added a linear decoding layer and used the logistic function to classify the activity of the decision unit into a binary perceptual choice (that is, clockwise or counterclockwise rotation of the target stimulus relative to the reference stimulus). Importantly, similar to previous psychophysical studies^7,21^, we systematically manipulated the level of input image noise (Fig. 1b). The network was trained on stimuli with multiple noise and contrast levels (see Methods for training details).

To evaluate the performance of the neural network, we assessed orientation discrimination accuracy as a function of stimulus contrast and noise (Fig. 1c,d) and further derived contrast thresholds as a function of image noise level (Fig. 1f, threshold versus noise (TvN) function). We found that training improved the network performance in this task in almost all stimulus contrast and noise conditions. The uniform downshift of TvN functions (Fig. 1f) is consistent with well-established human psychophysical results (replotted in Fig. 1e)^7,8^.

### VPL refines neural population representations in DCNN

**Fig. 2:**
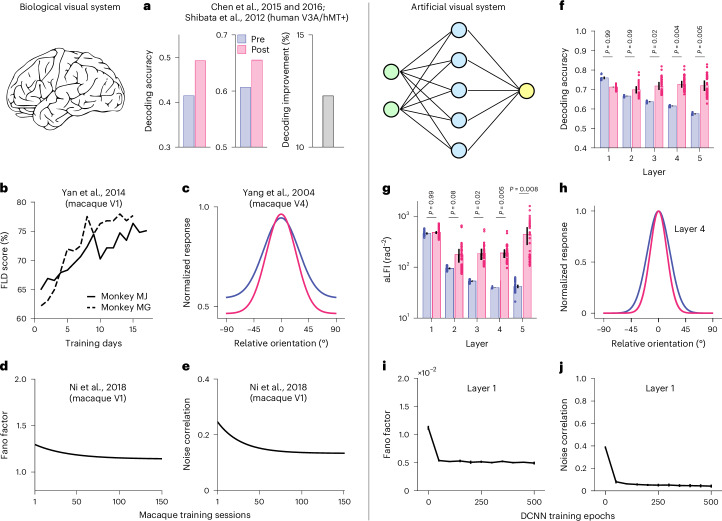
DCNN models reproduce empirical findings. **a**–**j**, Neural correlates of VPL in humans (**a**), monkeys (**b**–**e**) and our DCNN (**f**–**j**). Visual training improves stimulus decoding accuracy in related regions in the human brain (**a**) and decoding scores of Fisher’s linear discriminant (FLD) in monkey V1 (**b**). Visual training sharpens orientation tuning curves of neurons in monkey V4 (**c**) and also reduces Fano factors and interneuron noise correlations (**d** and **e**). Similar results are observed in the DCNN: network training also improves decoding accuracy in layers 3–5 (layers 1 and 2: one-sided paired *t*-test, *t*_(3)_ > −1.79, all *P* > 0.08; layers 3–5: one-sided paired *t*-test, *t*_(3)_ < −3.59, all *P* < 0.02; see full statistical results in Supplementary Table 1; **f**), and aLFI (total information in each layer divided by the number of units in that layer) in layers 3–5 (layers 1 and 2: one-sided paired *t*-test, *t*_(3)_ > −1.84, all *P* > 0.08; layers 3–5: one-sided paired *t*-test, all *t*_(3)_ < −3.47, all *P* < 0.02; see full statistical results in Supplementary Table 2; **g**). Training sharpens orientation tuning curves of units in layers 1–4 in the DCNN (results of layer 4 only are shown in **h**). Similar reduction of Fano factors and noise correlations are observed in the DCNN (results of layer 1 only are shown in **i** and **j**). The data shown in **h**–**j** are the median value across units in a layer. The results of all five layers are shown in Supplementary Fig. 1. Panels **a**–**e** are reproduced by the data points shown in the original papers. Data are presented as mean ± s.e.m., with error bars and error shadings in **f**–**j** represent the s.e.m. across four (*n* = 4) reference orientations (error shadings in **h** are small and barely visible).

We next sought to understand the effects of visual training on population representations in the network. We performed multivariate decoding analyses in each layer and found that training significantly improved decoding accuracy in later layers (Fig. 2f, layers 3–5; one-sided paired *t*-test, all *t*_(3)_ < −3.59, all *P* < 0.020; see full statistical results in Supplementary Table 1). More formally, we calculated linear Fisher information, a classical metric in computational neuroscience, to quantify how well the two stimuli can be discriminated on the basis of population responses (Methods). The amount of sensory information represented in later layers was indeed significantly enhanced by training (Fig. 2g, layers 3–5; one-sided paired *t*-test, all *t*_(3)_ < −3.47, all *P* < 0.018; see full statistical results in Supplementary Table 2). Such refined neural representation at the population level is consistent with the decoding results based on both cortical activity in humans^3,4,22^ and multiunit spiking activity in monkeys^13,14^.

### VPL changes response properties of individual units in DCNN

In addition to the population-level changes, we found that three key individual-level neural signatures of VPL as documented in the neurophysiological literature emerge naturally from the neural network training. First, training modestly sharpened the tuning curves of artificial neurons in layers 1–4 (Fig. 2h and Supplementary Fig. 1), a finding reported in several previous studies^9,10,23^. (Fig. 2c, but see also null results in ref. ^24^). Second, we observed a decrease in Fano factor of individual units in all five layers (Fig. 2i and Supplementary Fig. 1), a phenomenon indicating an increased SNR of individual neuronal responses in both humans^25^ and monkeys^11,23^ (Fig. 2d). The sharpened tuning curve and reduced Fano factor are also consistent with theoretical modelling^19^. Third, training reduced trial-by-trial noise correlations between units in all five layers (Fig. 2j and Supplementary Fig. 1), a finding also consistent with several empirical results in monkeys^11–14^. Critically, we also found that the reduction in noise correlation depended on tuning similarity. Learning reduced the noise correlations between units with similar tunings (that is, positive signal correlations) and increased the noise correlations between units with opposite tunings (that is, negative signal correlations) (Supplementary Fig. 2). Previous theoretical work has suggested that the former type of noise correlations is detrimental for information coding and the latter type is beneficial^15,16^. The pattern of reduced detrimental and increased beneficial noise correlations has been discovered with learning tasks in songbirds^26^ and with attention tasks in monkeys^27^.

In addition to these classical neurophysiological findings in VPL, our network also captures some important response properties of sensory neurons in the primate early visual system. First, the relationship between the Fano factor and orientation tuning of the artificial neurons bears strong resemblances to the empirical measures of V1 neurons in monkeys^28^ (Supplementary Fig. 2). Second, we found a positive relationship between signal correlation and noise correlation among artificial neurons in all layers (Supplementary Fig. 2). This relationship has also recently been documented as a ubiquitous phenomenon in both electrophysiological^29–31^ and human imaging^17,18,32^ studies.

Taken together, these results suggest that our DCNNs are powerful models and allow us to explore neurocomputational mechanisms that may be difficult to elucidate in empirical experiments. Here we focus on the qualitative similarities of learning-induced changes in DCNN and in certain brain regions. However, we did not attempt to claim one-on-one mapping between DCNN layers and brain regions because this requires one to build precise encoding models.

### Four mechanisms and the neural geometry approach of VPL

**Fig. 3:**
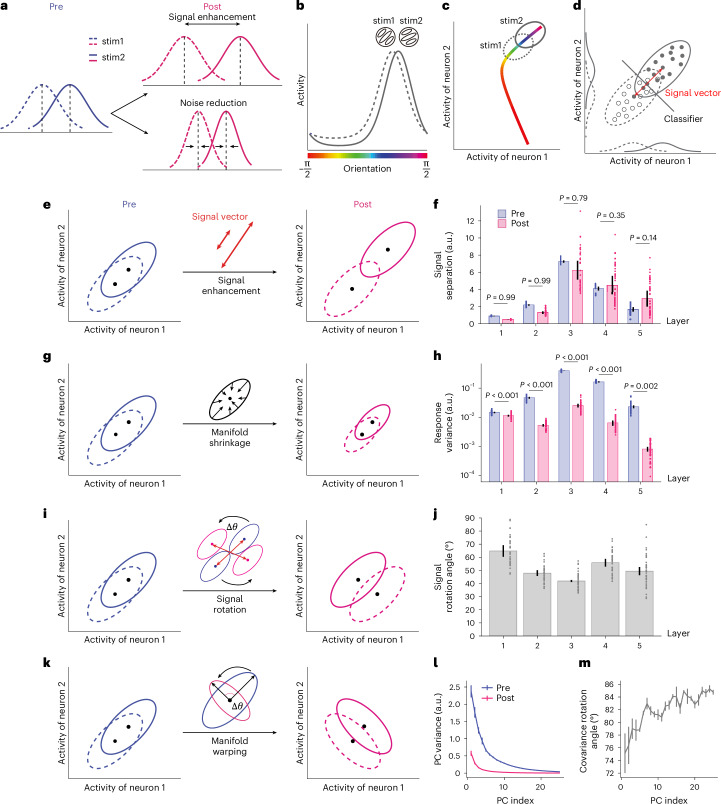
Four possible mechanisms of VPL in neural populations. **a**, To enhance sensory discriminability, the classical signal detection theory posits that signal enhancement predicts enlarged distances between two mean values while noise reduction predicts reduced variance of the two stimulus response distributions (stim1 and stim2). **b**, Stimulus orientation as a continuous stimulus variable can evoke high-dimensional population responses. **c**, If we continuously sweep the orientation value, the mean of population responses forms a closed-form ring in a high-dimensional neural space with dimensions equal to the number of units. The mean population responses to the two stimuli in a discrimination task are two points on the manifold. **d**,**e**, In realistic population responses, the trial-by-trial population responses to the two stimuli form two high-dimensional response distributions (that is, neural manifolds, **d**). The manifolds look elliptical rather than spherical due to pairwise noise correlations between units. In this high-dimensional neural space, the signal enhancement mechanism predicts an increased Euclidean distance (that is, signal separation, **e**) between two high-dimensional response distributions. **f**, However, no significant increase in signal separation is observed in any of the five layers (signal separation decreases in the first two layers; one-sided paired *t*-test, all *t*_(3)_ > −1.27, all *P* > 0.146, all BF_10_ <1.46; see full statistical results in Supplementary Table 3). **g**, The manifold shrinkage mechanism predicts reduced variance of the two neural manifolds. **h**, This is observed in all five layers (one-sided paired *t*-test, all *t*_(3)_ > 8.39, all *P* < 0.002; see full statistical results in Supplementary Table 4). **i**, The signal rotation mechanism predicts that the positions of the centroid (that is, mean) of the two manifolds are changed by training. **j**, The rotation angle ranges from approximately 50° to 70° in all five layers. **k**, The manifold warping mechanism predicts that training changes the shape of noise correlations. **l**, Indeed, training mostly reduces the variance of the high-variance principal components of the population responses. The principal components (showing only components that account for >99% of the total variance) are ranked from high to low variance. **m**, The directions of the principal components rotate from pre- to post-test. Data are presented as mean ± s.e.m., with error bars and error shadings in **f**–**m** representing the s.e.m. across four (*n* = 4) reference orientations.

How would improved sensory discrimination manifest in high-dimensional population responses? In the simplified one-dimensional scenario (Fig. 3a), the classical signal detection theory posits that better sensory discrimination can be achieved by either increasing the distance between the means (that is, signal enhancement) and/or decreasing the variance (that is, noise reduction) of the two response distributions. In multivariate population responses, the two stimuli to be discriminated instead generate two multivariate response distributions (that is, neural manifold) in a high-dimensional neural space whose dimension corresponds to the number of units in a population (Fig. 3b,c). In a simplified visualization in a two-dimensional space (Fig. 3d), the two distributions are elliptical due to noise correlations between units. We refer to the vector connecting the mean of the two distributions as the signal vector and its modulus length (that is, the Euclidean distance between the two manifold centroids) as the signal separation.

In the high-dimensional neural space, our neural geometry approach of VPL proposes that visual training improves sensory discrimination by shaping some fundamental geometric properties of the neural manifolds. Here, under this approach, there exist only four possible mechanisms to further separate two neural manifolds (equation (4) in Methods). First, according to the classical signal detection theory, the signal enhancement mechanism predicts an increased Euclidean distance between the centroids of the two neural manifolds (Fig. 3e). However, we found that the signal separation between the two manifolds did not significantly increase with learning in all five layers, and even slightly decreased in the first two layers (Fig. 3f; one-sided paired *t*-test, all *t*_(3)_ > −1.27, all *P* > 0.146, all Bayes factor BF_10_ <1.46; see full statistical results in Supplementary Table 3). Second, the manifold shrinkage mechanism predicts that visual training reduces the trial-by-trial response variance of units, thereby reducing the size of the manifolds (Fig. 3g). This is what we found in all five layers (Fig. 3h; one-sided paired *t*-test, all *t*_(3)_ > 8.39, all *P* < 0.002; see full statistical results in Supplementary Table 4). We further included two previously overlooked mechanisms that can only occur in high-dimensional neural space and increase manifold discriminability. In the third mechanism, although visual training did not increase signal separation, it may change the relative positions of the centroids of the two manifolds and consequently increase discriminability due to the elliptical shape of the manifolds (Fig. 3i). Interestingly, we found that the signal vectors in each layer were rotated by ~50–70° after training (Fig. 3j). We call this mechanism signal rotation. Fourth, visual training can warp the shapes of the high-dimensional neural manifolds while keeping the size of the manifolds unchanged. As indicated by the change of covariance structure, we found that visual training systematically warped the shape (that is, covariance structures) of the high-dimensional neural manifolds (Fig. 3k–m). We refer to this mechanism as manifold warping. Note that manifold warping includes both the changes in correlation structures and the redistribution of variances across individual units, while holding the total variance constant. It is manifold shrinkage that attenuates the total variance.

### Information-theoretic analyses quantified mechanisms of VPL

**Fig. 4:**
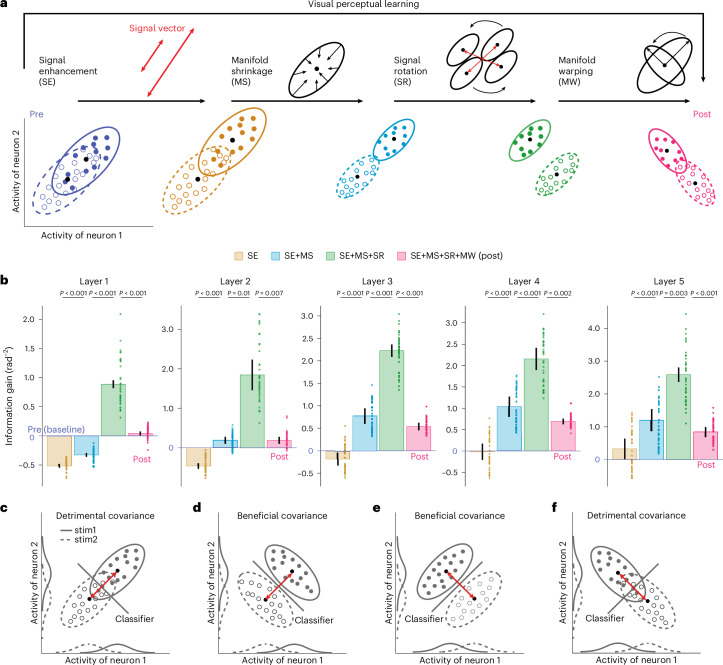
Information decomposition in neural populations. **a**, The effects of four mechanisms on population representations are decomposed into four distinct steps. **b**, The effects on information gain by sequentially adding each of the four mechanisms in each layer. For example, the increase in height from the brown to the blue bars indicates the positive contribution of manifold shrinkage to encoded stimulus information. Manifold shrinkage significantly increases the information (one-sided paired *t*-test, all *t*_(3)_ > 12.1, all *P* < 0.001); signal rotation significantly increases the information (one-sided paired *t*-test, all *t*_(3)_ > 5.2, all *P* < 0.006); manifold warping significantly decreases the information (one-sided paired *t*-test, all *t*_(3)_ > 4.3, all *P* < 0.01). See full statistical results in Supplementary Table 5. **c**–**f**, Strong interaction effects between covariance and signal vector. For distributions with identical covariance (**c** and **e**; **d** and **f**), detrimental (**c** or **f**) or beneficial (**d** or **e**) effects on discriminability are possible, depending on the signal vector. Similarly, the effects of the signal vector also depend on its relative geometry to the axis of covariance. Data are presented as mean ± s.e.m, with error bars and error shadings in **b** representing the s.e.m. across four (*n* = 4) reference orientations.

Given the four possible mechanisms (that is, signal enhancement, manifold shrinkage, signal rotation and manifold warping) and their complex interaction effects, how can we delineate their respective contributions to improved population representations? Here we use linear Fisher information to quantify manifold separability. Besides, we introduce a stepwise approach to further disentangle the respective contributions of the four possible mechanisms. Specially, their respective contributions are assessed by sequentially allowing only one mechanism to occur and quantifying its endowed changes in the linear Fisher information of whole populations (Fig. 4a). For example, as shown in Fig. 4, we first calculate how much information is enhanced by considering only the signal enhancement scenario, then by considering both signal enhancement and manifold shrinkage, and so on until all four mechanisms are included.

Interestingly, we found that the effect of signal enhancement is minimal in all five layers. This mechanism even reduces stimulus information in layers 1 and 2. This is consistent with the reduced Euclidean distance in the first two layers (Fig. 3f). Manifold shrinkage enhances stimulus information in almost all layers. Interestingly, we found that signal rotation appears to enhance stimulus information (Fig. 4b, green bars). This is because rotation of the signal vectors disrupts their relative parallelism to the covariance direction at pre-test, making them more orthogonal. Such changes increase the apparent information. However, the effect becomes minimal when manifold warping is further considered (Fig. 4b, magenta bars) because visual training also warps the covariance direction to realign it with the post-test signal vector, thereby reducing stimulus information (see more explanations in Supplementary Note 1 and full statistical results in Supplementary Table 5).

Taken together, we propose an interpretable and quantitative neural geometry approach of VPL where visual training refines the geometry of representations in a high-dimensional neural space. Using this approach, we found that three of four possible mechanisms occur in VPL. Most importantly, we found that manifold shrinkage in population responses was the key mechanism underlying the improved population representations induced by visual training in the DCNN. However, all above findings are the results of theoretical modelling using our DCNN model of orientation discrimination learning. Several predictions here have never been tested or reported in empirical studies. In the rest of ‘Results’, we tested these predictions across diverse tasks, measurement modalities and species.

### Motion direction discrimination learning in DCNN

The above analyses focus only on one classical VPL task—orientation discrimination and a specific neural network structure—a six-layer convolutional neural network. In this section, we switch to motion VPL—another sensory domain that is also widely used in psychophysical^33,34^, human imaging^3,4^ and neurophysiological studies^35^. Importantly, motion VPL involves the processing of both spatial and temporal signals rather than merely static spatial information in orientation learning. Similarly, we inherited the first six layers of the pretrained C3D network^36^ and trained the neural network to perform a motion direction discrimination task commonly used in psychophysics (see Methods for stimulus and training details).

**Fig. 5:**
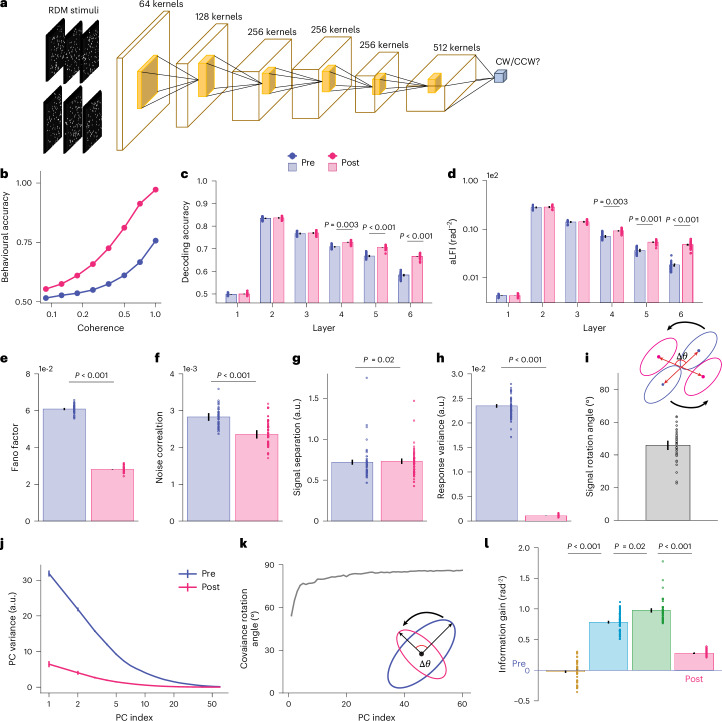
DCNN modelling of motion VPL. **a**, The DCNN of motion VPL uses 3D convolutions to process video stimuli. Here we simplify the four-dimensional feature maps in each convolutional layer and show them as 3D maps only for illustration purposes. **b**–**d**, Training improves DCNN direction discrimination performance (**b**), decoding accuracy (**c**; layers 4–6: one-sided paired *t*-test, all *t*_(3)_ < −7.14, all *P* < 0.028; see full statistical results in Supplementary Table 6) and aLFI (**d**; layers 4–6: one-sided paired *t*-test, all *t*_(3)_ < −7.22, all *P* < 0.003;; see full statistical results in Supplementary Table 7). **e**,**f**, For single-unit analyses, motion direction discrimination training also reduces the Fano factor (**e**; one-sided paired *t*-test, *t*_(3)_ = 57.58, *P* < 0.001, one-sided 95% CI 3.1 × 10^−2^ to ∞, Cohen’s *d* = 38.75) and noise correlation (**f**; one-sided paired *t*-test, *t*_(3)_ = 42.84, *P* < 0.001, one-sided 95% CI 4.4 × 10^−4^ to ∞, Cohen’s *d* = 2.19) in layer 6. **g**,**i**, Similar to orientation discrimination training, motion direction discrimination training does not significantly enhance signal separation (**g**; one-sided paired *t*-test, *t*_(3)_ = −0.98, *P* = 0.198, one-sided 95% CI −∞ to 1.7 × 10^−2^, Cohen’s *d* = −0.19, BF_10_ 1.22) but rotates the position of the two distributions in layer 6 (**i**). **h**, Importantly, training clearly reduces the response variance in layer 6 (one-sided paired *t*-test, *t*_(3)_ = 59.05, *P* < 0.001, one-sided 95% CI 2.2 × 10^−2^ to ∞, Cohen’s *d* = 43.89). **j**,**k**, Specifically, training reduces the variance of the high-variance PCs (**j**) and rotates the directions of all PCs (**k**), indicating a significant effect of manifold warping in layer 6. **l**, The pattern of information gain associated with the four possible mechanisms is consistent with that of orientation discrimination training (one-sided paired *t*-test, *t*_(3)_ = 76.0, *P* < 0.001 for manifold shrinkage, *t*_(3)_ = 3.80, *P* = 0.02 for signal rotation, *t*_(3)_ = 17.7, *P* < 0.001 for manifold warping). See results for all six layers in Supplementary Fig. 3. Data are presented as mean ± s.e.m., with error bars and error shadings in **c**–**l** representing the s.e.m. across four (*n* = 4) reference directions. Note that some error bars are very small and barely visible.

In the motion DCNN, we found similar mechanisms as in the orientation discrimination learning task. First, motion direction discrimination training improved the behavioural performance of the network (Fig. 5b). Second, training also enhanced decoding accuracy and averaged linear Fisher information (aLFI) in later layers (Fig. 5c,d; layers 4–6: for decoding accuracy: one-sided paired *t*-test, all *t*_(3)_ < −7.14, all *P* < 0.02; for aLFI: one-sided paired *t*-test, all *t*_(3)_ < −7.22, all *P* < 0.003; see full statistical results in Supplementary Tables 6 and 7), suggesting that such training refines stimulus representation at the population level. Third, the effects of motion direction discrimination training on individual units in layer 6 are also pronounced (see results for all six layers in Supplementary Fig. 3). We found that training reduced Fano factor (Fig. 5e; one-sided paired *t*-test, *t*_(3)_ = 57.58, *P* < 0.001, one-sided 95% confidence interval (CI) 3.1 × 10^−2^ to ∞, Cohen’s *d* = 38.75) and noise correlations (Fig. 5f; one-sided paired *t*-test, *t*_(3)_ = 42.84, *P* < 0.001, one-sided 95% CI 4.4 × 10^−4^ to ∞, Cohen’s *d* = 2.19). Fourth, training did not significantly improve signal separation (Fig. 5g; one-sided paired *t*-test, *t*_(3)_ = −0.98, *P* = 0.198, one-sided 95% CI −∞ to 1.7 × 10^−2^, Cohen’s *d* = −0.19, BF_10_ 1.22) but markedly reduced response variance (Fig. 5h; one-sided paired *t*-test, *t*_(3)_ = 59.05, *P* < 0.001, one-sided 95% CI 2.2 × 10^−2^ to ∞, Cohen’s *d* = 43.89). In addition, motion direction discrimination training also induced two previously overlooked mechanisms: signal rotation (Fig. 5i) and manifold warping (Fig. 5j,k). Most importantly, the four mechanisms induced by the training had similar respective contributions to population representations (Fig. 5l).

### Motion direction discrimination learning in the human brain

**Fig. 6:**
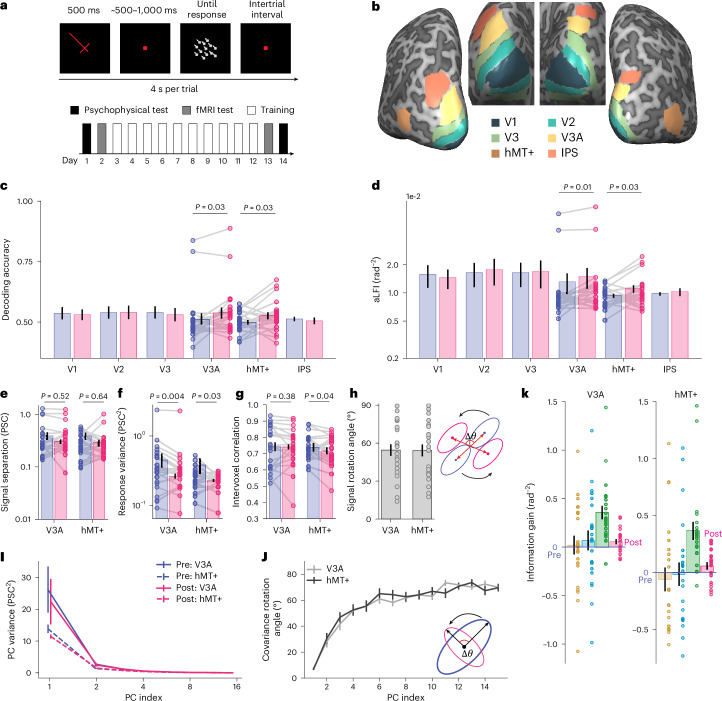
Motion VPL induces neural geometry changes in the human brain. **a**,**b**, Trial diagram and training paradigm (**a**), and ROIs in a typical subject (**b**). **c**,**d**, Motion direction discrimination training in humans significantly improves decoding accuracy (**c**; for V3A: one-sided paired *t*-test, *t*_(21)_ = −2.01, *P* = 0.029, one-sided 95% CI −∞ to −3.7 × 10^−3^, Cohen’s *d* = −0.25; for hMT+: one-sided paired *t*-test, *t*_(21)_ = −1.95, *P* = 0.032, one-sided 95% CI −∞ to −3.3 × 10^−3^, Cohen’s *d* = −0.50) and aLFI (**d**; for V3A: one-sided paired *t*-test, *t*_(21)_ = −2.36, *P* = 0.014, one-sided 95% CI −∞ to −5.0 × 10^−4^, Cohen’s *d* = −0.11; for hMT+: one-sided paired *t*-test, *t*_(21)_ = −1.99, *P* = 0.030, one-sided 95% CI −∞ to −2.3 × 10^−4^, Cohen’s *d* = −0.47) in areas V3A and hMT+, a finding consistent with several existing fMRI studies of motion VPL. Note that the four data points in V3A appear as outliers in **c** and **d**, but the results still hold if these data points are removed. **e**–**g**, Motion direction discrimination training does not significantly change signal separation in V3A and hMT+ (**e**; for V3A: one-sided paired *t*-test, *t*_(21)_ = 0.06, *P* = 0.526, one-sided 95% CI −∞ to 5.3 × 10^−2^, Cohen’s *d* = 0.01, BF_10_ 0.45; for hMT+: one-sided paired *t*-test, *t*_(21)_ = 0.36, *P* = 0.639, one-sided 95% CI −∞ to 9.5 × 10^−2^, Cohen’s *d* = 0.09, BF_10_ 0.47) but reduces voxel response variance in V3A and hMT+ (**f**; for V3A: one-sided paired *t*-test, *t*_(21)_ = 2.87, *P* = 0.004, one-sided 95% CI 2.9 × 10^−2^ to ∞, Cohen’s *d* = 0.16; for hMT+: one-sided paired *t*-test, *t*_(21)_ = 1.97, *P* = 0.031, one-sided 95% CI 4.6 × 10^−3^ to ∞, Cohen’s *d* = 0.46) and intervoxel noise correlations in hMT+ (**g**; one-sided paired *t*-test, *t*_(21)_ = 1.90, *P* = 0.035, one-sided 95% CI 2.5 × 10^−3^ to ∞, Cohen’s *d* = 0.31). **h**–**j**, Similar to the motion DCNNs, motion direction discrimination training in humans also rotates stimulus distributions (**h**), reduces the variance of high-variance PCs (**i**) and warps the covariance directions (**j**). **k**, The patterns of information gain associated with the four mechanisms are consistent with those in the DCNNs of both orientation and motion VPL. The unit PSC represents percent signal change of BOLD signals. Individual data points represent the human subjects. Data are presented as mean ± s.e.m., with error bars in all panels representing the s.e.m. across subjects (*n* = 22). *P < 0.05, **P < 0.01.

The converging results in the DCNNs of orientation and motion direction discrimination, and the remarkable agreement between our DCNNs and existing empirical neuroscientific findings, support the biological plausibility of our DCNNs. However, it remains unknown whether these predictions are present only in the DCNNs and have no biological basis in the brain. To address this question, we analysed BOLD responses in the cortex of human subjects before and after they were trained on a motion direction discrimination task (Fig. 6a, ref. ^37^). Twenty-two human subjects participated in the motion VPL study. Subjects were trained for 10 days on a fine-direction discrimination task, and psychophysical and functional magnetic resonance imaging (fMRI) tests were performed before and after training.

We identified the early visual areas (V1–V3), the motion-selective regions (V3A and hMT+) and the decision region (intraparietal sulcus, IPS) using independent functional localizer experiments (Fig. 6b). We estimated single-trial responses of voxels in these regions and then performed decoding analyses in these predefined regions, finding that motion training significantly enhanced decoding accuracy (Fig. 6c; V3A: one-sided paired *t*-test, *t*_(21)_ = −2.01, *P* = 0.029, one-sided 95% CI −∞ to −3.7 × 10^−3^, Cohen’s *d* = −0.25; hMT+: one-sided paired *t*-test, *t*_(21)_ = −1.95, *P* = 0.032, one-sided 95% CI −∞ to −3.3 × 10^−3^, Cohen’s *d* = −0.50) and aLFI (Fig. 6d) in areas V3A and hMT+ (V3A: one-sided paired *t*-test, *t*_(21)_ = −2.36, *P* = 0.014, one-sided 95% CI −∞ to −5.0 × 10^−4^, Cohen’s *d* = −0.11; hMT+: one-sided paired *t*-test, *t*_(21)_ = −1.99, *P* = 0.030, one-sided 95% CI −∞ to −2.3 × 10^−4^, Cohen’s *d* = −0.47), a result consistent with several human fMRI studies on motion VPL^3,4,22^.

We further investigated the coding principles in areas V3A and hMT+ and repeated the above analyses of DCNNs on fMRI data. Note that here we performed the same analyses on voxels instead of artificial neurons in DCNNs. Consistent with the predictions of the DCNNs, motion direction discrimination training in humans did not increase signal separation (Fig. 6e, V3A: one-sided paired *t*-test, *t*_(21)_ = 0.06, *P* = 0.526, one-sided 95% CI −∞ to 5.3 × 10^−2^, Cohen’s *d* = 0.01, BF_10_ 0.45; hMT+: one-sided paired *t*-test, *t*_(21)_ = 0.36, *P* = 0.639, one-sided 95% CI −∞ to 9.5 × 10^−2^, Cohen’s *d* = 0.09, BF_10_ 0.47) but markedly reduced voxel response variance (Fig. 6f) in both areas (V3A: one-sided paired *t*-test, *t*_(21)_ = 2.87, *P* = 0.004, one-sided 95% CI 2.9 × 10^−2^ to ∞, Cohen’s *d* = 0.16; hMT+: one-sided paired *t*-test, *t*_(21)_ = 1.97, *P* = 0.031, one-sided 95% CI 4.6 × 10^−3^ to ∞, Cohen’s *d* = 0.46). Motion direction discrimination training also significantly reduced intervoxel correlations in hMT+ (Fig. 6g; one-sided paired *t*-test, *t*_(21)_ = 1.90, *P* = 0.035, one-sided 95% CI 2.5 × 10^−3^ to ∞, Cohen’s *d* = 0.31). The mechanism of signal rotation was also evident, as indicated by the average ~55° rotation of the signal vectors in both areas (Fig. 6h). In addition, training warped the magnitude and direction of the covariance (Fig. 6i,j). Most importantly, the respective contributions of these four mechanisms in both brain regions were similar to the pattern in the DCNNs (Fig. 6k).

### Contrast discrimination learning in monkey V4

Voxel responses in fMRI studies reflect macroscopic brain activity that aggregates the responses of ~300,000–50,000 neurons^38^. It remains unclear whether the mechanisms we have discovered so far also exist at the local circuit level of single neurons or small clusters of neurons. To our knowledge, these predictions based on our neural network models have not been systematically tested using intracranial recording.

**Fig. 7:**
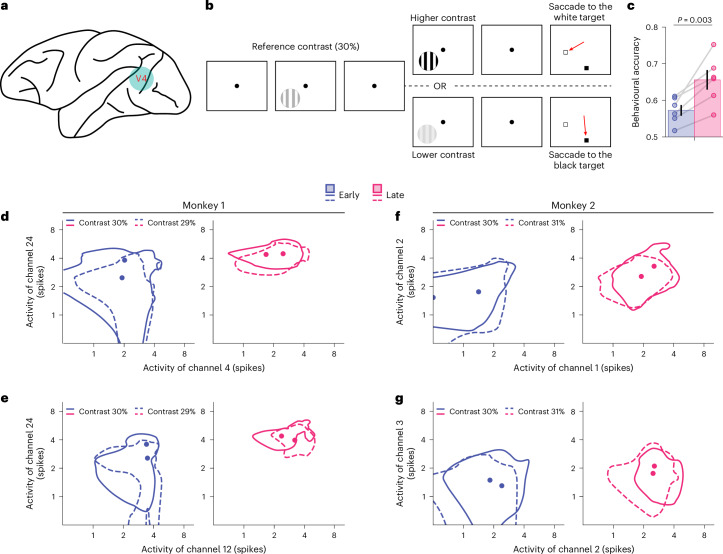
Single-unit analyses of contrast discrimination learning in monkey V4. **a**,**b**, We analysed population responses in area V4 (**a**) of two monkeys while they were trained on a fine contrast discrimination task (**b**). The first four and last four training sessions were grouped as pre- and the post-test conditions, respectively. Contrast discrimination training significantly improved behavioural performance from the early to late stage of training (**c**; one-sided paired *t*-test, *t*_(5)_ = −4.61, *P* = 0.003, Cohen’s *d* = −1.57). **c**, All individual data points represent the six target contrast conditions (27%, 28%, 29%, 31%, 32% and 33%; the reference contrast is 30%). Each point is averaged over the two monkeys. See plots for individual monkeys in Supplementary Fig. 4. Data are presented as mean ± s.e.m., with error bars indicate the s.e.m. across the six conditions (*n* = 6). **d**–**g**, The full width at half maximum of the response distributions of four pairs of channels at pre- and post-test (**d** and **e** for monkey 1 and **f** and **g** for monkey 2). The solid lines represent 30% reference contrast, and the dashed lines represent 29% and 31% target contrast in monkey 1 and monkey 2, respectively. These results show that learning systematically changes the geometries of the multivariate responses.

To further test our hypotheses on neuronal spiking activity, we analysed the population responses of V4 neurons in two monkeys (Fig. 7a) at the early stage and at the late stage of learning to perform a fine-contrast discrimination task (Fig. 7b, ref. ^14^). In this task, each monkey was presented sequentially with two identical Gabor patches with different contrast levels. The contrast of the reference (that is, the first) stimulus was always fixed at 30%, and the contrast of the target (that is, the second) stimuli varied systematically near the reference contrast (that is, 27%, 28%, 29%, 31%, 32% and 33%). This contrast discrimination training significantly improved behavioural performance (Fig. 7c; one-sided paired *t*-test, *t*_(5)_ = −4.61, *P* = 0.003, one-sided 95% CI −∞ to −4.7 × 10^−2^, Cohen’s *d* = −1.57). Most importantly, responses of multiple channels were continuously recorded via chronically implanted electrodes in area V4 (29 and 20 channels for monkeys 1 and 2, respectively) throughout training (21 and 23 training sessions for the two monkeys, respectively). This continuous multiunit recording is the key to disentangling population-level changes associated with VPL.

**Fig. 8:**
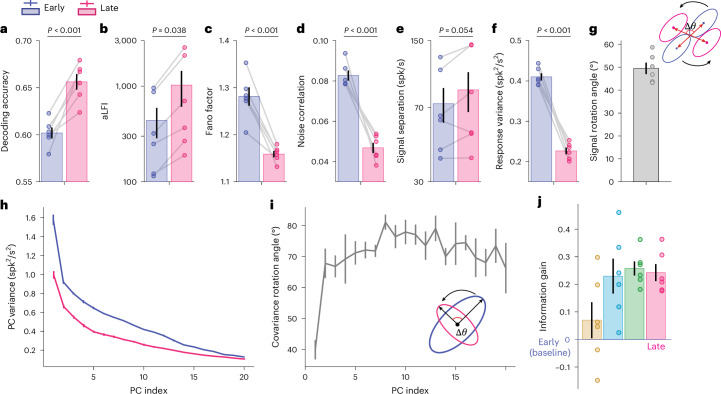
Population activity analyses of contrast discrimination learning in monkey V4. **a**,**b**, Contrast discrimination training significantly enhanced stimulus information at the population level (for decoding accuracy: one-sided paired *t*-test, *t*_(5)_ = −6.03, *P* < 0.001, one-sided 95% CI −∞ to −3.6 × 10^−2^, Cohen^’^s *d* = −3.10 (**a**); for aLFI: one-sided paired *t*-test, *t*_(5)_ = −2.21, *P* = 0.039, one-sided 95% CI −∞ to −52, Cohen’s *d* = −0.76 (**b**)). **c**–**f**, Consistent with VPL in the DCNNs and the human brain, training monkeys on a contrast discrimination task reduced Fano factors (**c**; one-sided paired *t*-test, *t*_(5)_ = 7.28, *P* < 0.001, one-sided 95% CI 8.8 × 10^−2^ to ∞, Cohen’s *d* = 3.43), noise correlations (**d**; one-sided paired *t*-test, *t*_(5)_ = 7.46, *P* < 0.001, one-sided 95% CI 2.6 × 10^−2^ to ∞, Cohen’s *d* = 5.80) and response variance (**f**; one-sided paired *t*-test, *t*_(5)_ = 13.24, *P* < 0.001, one-sided 95% CI 1.6 × 10^−1^ to ∞, Cohen’s *d* = 8.70) but had no significant effect on signal separation (**e**; one-sided paired *t*-test, *t*_(5)_ = −1.957, *P* = 0.054, one-sided 95% CI −3.7 × 10^−1^ to ∞, Cohen’s *d* = −0.30, BF_10_ 2.41). **g**–**i**, We also found evidence for signal rotation (**g**) and manifold warping (**h** for PC variance and **i** for PC rotation). **j**, The stepwise information analyses also show the similar pattern of the four mechanisms. The unit ‘spk/s’ indicates the number of spikes per second (that is, firing rate). We calculate aLFI and information gain using stimulus contrast as decimal values (that is, 0.29), so they have arbitrary units. Each point is averaged over the two monkeys. See plots for individual monkeys in Supplementary Fig. 4. Data are presented as mean ± s.e.m., with error bars indicating the s.e.m. across the six conditions (*n* = 6).

We used the above analyses (previously applied to DCNNs and human fMRI data) and applied them to the monkey V4 responses, and again found highly consistent results (see results of each monkey in Supplementary Fig. 4). First, contrast discrimination training significantly improved stimulus information at the population level (Fig. 8a,b; decoding accuracy: one-sided paired *t*-test, *t*_(5)_ = −6.03, *P* < 0.001, one-sided 95% CI −∞ to −3.6 × 10^−2^, Cohen’s *d* = −3.10; aLFI: one-sided paired *t*-test, *t*_(5)_ = −2.21, *P* = 0.039, one-sided 95% CI −∞ to −52, Cohen’s *d* = −0.76). Second, at the individual level, contrast discrimination training also significantly reduced Fano factors (Fig. 8c; one-sided paired *t*-test, *t*_(5)_ = 7.28, *P* < 0.001, one-sided 95% CI 8.8 × 10^−2^ to ∞, Cohen’s *d* = 3.43) and noise correlations (Fig. 8d; one-sided paired *t*-test, *t*_(5)_ = 7.46, *P* < 0.001, one-sided 95% CI 2.6 × 10^−2^ to ∞, Cohen’s *d* = 5.80), consistent with several existing findings. Interestingly, while the trial-by-trial variance was significantly reduced after training (Fig. 8f; one-sided paired *t*-test, *t*_(5)_ = 13.24, *P* < 0.001, one-sided 95% CI 1.6 × 10^−1^ to ∞, Cohen’s *d* = 8.70), no apparent change in signal separation was observed (Fig. 8e; one-sided paired *t*-test, *t*_(5)_ = −1.957, *P* = 0.054, one-sided 95% CI −3.7 × 10^−1^ to ∞, Cohen’s *d* = −0.30, BF_10_ 2.41), suggesting the predominant role of manifold shrinkage. Importantly, we again observed evidence for signal rotation (Fig. 8g) and manifold warping (Fig. 8h,i). The stepwise information analyses also qualitatively replicated the relative contributions of the four mechanisms to the total stimulus information encoded in the population (Fig. 8j).

## Discussion

It has been controversial whether single-unit properties such as sharpened tuning curves^9,10^ or reduction of noise correlations^11,12^ contribute to VPL. Our information-theoretic analysis on neural geometry suggested that, although these changes were indeed observed, they did not contribute significantly to the improved population representations associated with VPL. Rather, we found that the totally overlooked mechanism—the response variance of individual units (that is, manifold shrinkage)—is the primary contributor to the improved population representations associated with VPL. These results were further tested on DCNNs, human fMRI data and monkey neurophysiological data associated with different VPL tasks and brain regions.

Given the pronounced changes in tuning curves and noise correlations observed after training, why do they not contribute to VPL? Conventional approaches treat changes in tuning curves and in noise correlations as two independent factors mediating VPL. However, according to the neural geometry approach, the effects of tuning curve changes can be decomposed into two parts: signal enhancement independent of noise correlations and signal rotation interacting with noise correlations (equation (4) in Methods). We observed minimal contributions of signal enhancement to population representations. Although we observed the phenomena of signal rotation and manifold warping, their respective contributions appeared significant but their overall joint effects were minimal because their respective effects can cancel each other out (Supplementary Fig. 5).

Our finding that manifold shrinkage is the primary contributor to improved population representations is of unique significance in constraining the model of VPL. We note that the goal of perceptual learning is to produce more discriminable population representations such that downstream decision units can easily read out sensory information. However, deciphering the underlying format of discriminable representations is non-trivial because discriminable representations can be achieved by any or combinations of four possible mechanisms. The key contribution of our work lies in the systematic quantification of the four mechanisms. In manifold shrinkage, the total variance of the high-dimensional distributions is scaled down (that is, ${\underline{\lambda}}$ in equation (4) is reduced). In other words, the two stimulus distributions simply shrink to a smaller size (Fig. 4). Note that manifold shrinkage is independent of any tuning changes and noise correlation changes. We also emphasize that manifold shrinkage and manifold warping are two different mechanisms. In our approach, manifold warping redistributes the variance of the high-dimensional distributions in different directions (that is, ${\hat{\lambda }}_{i}$ and ${\xi }_{i}$ in equation (4) are changed) but, unlike manifold shrinkage, the total amount of variance remains unchanged. Thus, the shape of the two stimulus distributions is significantly warped. We thus emphasize manifold shrinkage as a marker of global population responses to differentiate it from trial-by-trial variability changes in single units.

Our neural geometry approach is consistent with the earlier applications of high-dimensional signal detection theory (MSDT) in psychophysics and systems neuroscience^39,40^. MSDT is a powerful tool for quantifying the discriminability of population representations. Although we also focus on the discriminability of population representations during the learning process, we extend this framework by conceptualizing MSDT as neural geometries to bridge changes in individual-level responses and changes in population representations. Specifically, changes in individual responses (for example, changes in tuning curves and/or noise correlations) are characterized as geometric transformations (for example, signal enhancement and manifold warping) of neural manifolds. These geometric transformations elucidate how discriminability in high-dimensional response distributions is enhanced. This geometric perspective enables experimentally testable predictions about learning effects of on neural manifolds, offering insights to adjudicate previous theories of VPL.

This high-dimensional geometric approach has been used in topics such as classification^41^, attention^42^ and neural coding^43^. The geometric similarities also predict perceptual similarities in humans^44^. A recent study^45^ found that such coordinates are not arbitrary, but privileged. The high-dimensional representational axes are highly consistent across different humans and even across different DCNNs. These representational axes lead to better readout or generalization abilities.

Our work unifies several important existing findings of VPL. First, it has long been hypothesized that noise reduction is an important mechanism of VPL^6,7,21^, but the exact underlying neural mechanisms remain elusive. Our work demonstrates that at least manifold shrinkage due to reduced trial-by-trial response variability is a viable mechanism to support noise reduction. Second, Bejjanki et al.^46^ built a biological neural network and, similar to our task, simulated the effects of orientation VPL on Gabor stimuli with different levels of image noise. The results showed that changes in orientation tuning curve have only modest effects on psychophysical TvN functions. Using a different network architecture (pretrained artificial DCNNs), our study replicated the finding of sharpened orientation-selective tuning curves reported and also showed that the effects of such tuning changes are modest. Our modelling here suggests that sharpened tuning curves do not necessarily lead to improved population codes, given that other aspects of population responses are also changed by learning. Third, most existing human imaging studies and single-unit studies on VPL have focused only on changes in population representations^3,4,22^ or changes in individual neurons^9,10^, respectively. Previous studies attempted to address the relationship between the two levels by projecting high-dimensional neural manifolds onto a one-dimensional optimal decision plane^13,22^. However, we argue that this approach is inadequate (see analytical derivations in Supplementary Note 2) and we should explicitly disentangle and quantify the effects of individual factors (see additional analysis in Supplementary Fig. 6).

It is noteworthy that our approach is based on the assumption that VPL is associated with changes in neuronal populations. However, we do not dismiss all neuron-level accounts for VPL. For example, VPL could be conceptualized as a search in neuronal space for the most informative neurons for the trained task. These neurons are not necessarily the ones most responsive to the trained stimuli or those that represent them most efficiently. For example, post-adaptation orientation discrimination in expert subjects has been shown to involve learning that the most informative channel/filter for discrimination is rotated about 10–20° away from the observed stimulus^47^. Similar results were observed in monkey neurons during training of VPL of orientation discrimination^9^. The specific rotation magnitude may depend on the tuning curves and noise properties of the neurons.

Our study still has several limitations that could be addressed by future studies. First, although DCNN has recently emerged as a promising computational framework for modelling, there still exist clear differences between DCNNs and biological visual systems. Our models here are all feedforward architectures and lack the component of top-down modulation. Top-down modulation is an important aspect of supervised training^48^ and particularly useful for considering within-trial neural dynamics^49^. Second, VPL can be achieved by unsupervised training^50^ or even pure mental imagery^51^. These learning regimes cannot be explained by current models. Third, this study examines only how VPL improves population codes of trained stimuli. It remains unclear how learning effects generalize to other untrained stimuli, which is recently proposed as a key question in VPL^52^. Fourth, it remains unclear the perceptual consequences predicted by our neural geometry approach, especially by each mechanism. To address this, we conducted thorough simulations of neural geometric changes and derived their predictions on perceptual detection and perceptual estimation tasks (Supplementary Note 3 and Supplementary Fig. 7), which could be further tested in future studies. Our framework also provides a theoretical foundation to understand neural underpinnings of generalization in future studies.

## Methods

### DCNN modelling of orientation VPL

#### Stimuli

The network was trained to discriminate whether a target stimulus was tilted 1° clockwise or counterclockwise relative to a reference stimulus. All reference stimuli in the orientation discrimination task were Gabor patterns (227 × 227 pixels; spatial frequency, 40 pixels per cycle; standard deviation of the Gaussian spatial envelope, 50 pixels). The stimuli were varied in contrast (0.1 to 1.0 in 0.1 increments) and image noise level (eight levels: 0.005, 1, 5, 10, 15, 30, 50 and 75). Similar to existing psychophysical studies^53^, the image noise level is defined as the fraction of pixels randomly selected and replaced by Gaussian noise with a standard deviation of 15 gray level units. To mimic intrinsic sensory noise, we also added Gaussian white noise (standard deviation 10) to each stimulus^19^. To match the spatial frequency of noise and signal, the size of the replaced pixels was set to be 8 × 8. Four reference orientations (35°, 55°, 125° and 145°) were used, and we trained ten DCNNs (ten different random seeds, see below) for each of the four reference orientations. This yields 40 DCNNs models of VPL.

#### Neural networks and training

A DCNN^20^ was used to simulate the orientation VPL. We retained the first five convolutional layers of the pretrained AlexNet and replaced its three fully connected layers with a single linear fully connected layer for perceptual choice. The network was configured in a Siamese fashion to perform the two-alternative forced-choice task: the same network was fed with both the target and the reference stimuli, producing two scalar outputs, ${h}_{{\mathrm{t}}}$ and ${h}_{{\mathrm{r}}}$, respectively. The network then made the final decision with a probability *p* (classification confidence) calculated by the sigmoid function

1 $$p=\frac{{{\mathrm{e}}}^{{h}_{{\mathrm{t}}}-{h}_{{\mathrm{r}}}}}{1+{{\mathrm{e}}}^{{h}_{{\mathrm{t}}}-{h}_{{\mathrm{r}}}}}.$$

The entire training procedure consisted of two distinct phases: the pretraining phase and the VPL phase. In the pretraining phase, the network was trained on full-contrast noiseless stimulus pairs to understand the task and to establish the pre-test baseline. In the VPL phase, the network was trained on stimulus pairs across all contrasts (ten levels) and noise levels (eight levels). The network was trained for 5,000 epochs in the pretraining phase and 500 epochs in the VPL phase using the stochastic gradient descent learning algorithm. The learning rate and the momentum were set to 1e^−5^ and 0.9, respectively. The parameters were updated to minimize the cross-entropy loss between the network outputs and the true stimulus labels. The initial parameters in the fully connected layer were set to zero, as in ref. ^19^, while those in the convolutional layers were taken directly from a pretrained AlexNet available at http://dl.caffe.berkeleyvision.org/bvlc_AlexNet.caffemodel. We trained one model for each of the four reference orientations, and the entire procedure was repeated ten times for each reference orientation to control for randomness. All model and training procedures were implemented using Python 3.10.9 conda environment, including pytorch 1.13.1, scikit-learn 1.2.0. Details of the full conda environment are provided via GitHub at https://github.com/Yu-AngCheng/neural_geometry_VPL.

#### Behavioural and neural changes

For each reference orientation, we used the stimuli with the same orientations in pre-/post-tests and in training phase. The only difference is that stimulus images were randomly generated in each trial. We derived the behavioural psychometric curves of the network before and after the VPL phase defined above. Specifically, the behavioural performance of the network was evaluated by measuring its classification confidence (equation (1)) at all 80 conditions (10 contrast levels × 8 noise levels) with 1,000 trials in each condition. The classification confidence of all 1,000 trials was averaged (Fig. 1c,d). The behavioural TvN curves (Fig. 1f) of the model were further derived for comparison with human psychophysical results. Specifically, for each noise level, a contrast threshold was obtained by interpolating accuracy–contrast psychometric curves at the accuracies of 55% and 70% for pre-test and post-test respectively.

To quantify the activity of artificial neurons, in each trial, the firing rate of each artificial neuron was measured as the output of local response normalization or rectified linear unit (ReLU) layers, averaged over all locations. All measurements were obtained by simulating 1,000 trials for better estimation. To ensure that units were truly driven by the stimuli, only units with a mean firing rate greater than 0.001 before and after training were included in the analyses^19^. To perform population decoding analyses, we trained a linear classifier on the firing rates of the artificial neurons to discriminate the target and the reference stimuli. The classifier was trained on half of the 1,000 simulated trials, while the other half served as the test dataset.

To characterize the response properties of individual units, we measured orientation-selective tuning curves by sweeping the orientation of high-contrast stimuli from 0° to 180°. The tuning curves were derived by averaging 100 simulated trials for each orientation. The resulting tuning curves were then smoothed with a 10° Gaussian kernel. To control the heterogenous response range across units, we then normalized the tuning curves of each unit by its maximum response and averaged the tuning curves across units to obtain the group-level tuning curves. The group-level tuning curves were then fitted with a Gaussian function and rescaled to ~0–1 for better comparison.

To calculate the Fano factor of each unit, we simulated 1,000 trials for each reference orientation. The Fano factor of each artificial neuron is defined as the ratio of the variance of the firing rate to its mean. Similarly, noise correlations between artificial neurons were calculated as the correlations between unit firing rates over the 1,000 simulated trials for each reference orientation. We took the median of the Fano factor across units in each layer to generate the data plot (Fig. 2i). We took the median of the lower triangle of the noise correlation matrix in each layer to generate the data plot (Fig. 2j). The error bars in Fig. 2i,j represent the standard errors across four reference orientations.

#### Linear Fisher information analyses

To understand how neural activation contributes to behavioural improvements, we applied linear Fisher information analysis to population responses. We considered the firing rates of the same groups of units under the reference and the target stimulus conditions as two distributions in a high-dimensional neural space. We refer to the signal vector as the vector connecting the mean of the two distributions. A signal vector is calculated as the difference between the mean firing rates of units to two stimuli. The signal separation is referred to as the modulus length of the signal vector, and the angle of the signal vectors before and after training is referred to as the signal rotation angle.

To measure how much information was contained in a layer per unit, we calculated the aLFI as follows:

2 $${{\mathrm{aLFI}}}=\frac{1}{n}\cdot\frac{{{{\mathrm{d}}f}}^{\,T}{\overline{\Sigma }}^{-1} {{\mathrm{d}}f}}{{\Delta {\rm{\theta }}}^{2}}$$

3 $$\begin{array}{ccc}\bar{\Sigma }\,=\,\frac{{\Sigma }_{1}+{\Sigma }_{2}}{2}\\\qquad\qquad\qquad\quad\,\,\,\;\;=\,\frac{{V}_{1}^{T}{C}_{1} {V}_{1}+{V}_{2}^{T}{C}_{2}{V}_{2}}{2},\end{array}$$

where $n$ is the number of units in a layer, Δ*θ* is the separation between the target stimulus and the reference stimulus (that is, 1°), ${{\mathrm{d}}f}$ is the signal vector, $\overline{\Sigma }$ is the mean of the covariance matrices (that is, ${\Sigma }_{1}$ and ${\Sigma }_{2}$) of units responding to the two stimuli, $V$ is a diagonal matrix with the variance of the units as the diagonal terms, and $C$ is the correlation matrix of the population with all diagonal elements equal to 1.

To further elaborate on the potential mechanisms of the improved LFI, we performed an eigendecomposition on the covariance matrix $\overline{\Sigma }$, where we obtained *λ*_*i*_, the eigenvalue of $\bar{\Sigma }$, and ${\xi }_{i}$, its corresponding normalized eigenvector. The aLFI can be rewritten as follows:

4 $$\begin{array}{ccc}{{\mathrm{aLFI}}}\,=\,\frac{1}{n{\Delta {\rm{\theta }}}^{2}} \mathop{\sum }\limits_{i=1}^{N}\frac{{\left({{\mathrm{d}}f}^T {\xi }_{i}\right)}^{2}}{{\lambda }_{i}}\\\qquad\qquad\quad\,\,\,\,\,\,\,=\,\frac{1}{n{\Delta {\rm{\theta }}}^{2}}\frac{{\left|{{\mathrm{d}}f}\,\right|}^{2}}{{\underline{\lambda}}} \mathop{\sum }\limits_{i=1}^{N}\frac{{\left({{\hat{{\mathrm{d}}f}}^T}{\xi }_{i}\right)}^{2}}{{\hat{\lambda}}_{i}},\end{array}$$

where ${\underline{\lambda}}$ is the mean variance, and ${\lambda }_{i}={\underline{\lambda}}\times {\hat{\lambda }}_{i}$. ${\hat{{\mathrm{d}}f}}=\frac{{{\mathrm{d}}f}}{{|{\mathrm{d}}f\,|}}$ is the unit vector with length of 1 and direction as the same as the signal vector ${df}$. According to equation (4), we disentangled the potential mechanisms of improved LFI into four subparts: signal enhancement, reflected by the modulus length ${|{\mathrm{d}}f|}$; manifold shrinkage, reflected by the mean variance of $\bar{\lambda }$; signal rotation, reflected by the direction of the signal vector ${{\mathrm{d}}f}$; and manifold warping, reflected by the relative angle of both ${\xi }_{i}$ and ${\hat{\lambda }}_{i}$. We applied a stepwise approach to assess their respective contributions by sequentially allowing only one mechanism to occur and calculating the resulting changes in aLFI. Specifically, we first calculated aLFI at pre-test as

5 $${\mathrm{aLFI}}_{{{\mathrm{pre}}}}=\frac{1}{n{\Delta {\rm{\theta }}}^{2}} \frac{{\left|{\mathrm{d}}{f}_{{{\mathrm{pre}}}}\right|}^{2}}{{{\underline{\lambda}}}_{{{\mathrm{pre}}}}} \mathop{\sum }\limits_{i=1}^{N}\frac{{\left(\frac{{{\mathrm{d}}{f}_{{{\mathrm{pre}}}}}^T}{\left|{\mathrm{d}}{f}_{{{\mathrm{pre}}}}\right|} {\xi }_{i}^{{\,{\mathrm{pre}}}}\right)}^{2}}{{\hat{\lambda }}_{i}^{{{\mathrm{pre}}}}}.$$

Considering only the effect of signal enhancement, we can calculate its effect as

6 $${\mathrm{aLFI}}_{{{\mathrm{se}}}}=\frac{1}{n{\Delta {\rm{\theta }}}^{2}}\frac{{\left|{\mathrm{d}}{f}_{{{\mathrm{post}}}}\right|}^{2}}{{{\underline{\lambda}}}_{{{\mathrm{pre}}}}} \mathop{\sum }\limits_{i=1}^{N}\frac{{\left(\frac{{{\mathrm{d}}{f}_{{{\mathrm{pre}}}}}^T}{\left|{\mathrm{d}}{f}_{{{\mathrm{pre}}}}\right|} {\xi }_{i}^{{\,{\mathrm{pre}}}}\right)}^{2}}{{\hat{\lambda }}_{i}^{{{\mathrm{pre}}}}}.$$

Note that the only difference here is that the ${\left|{\mathrm{d}}{f}_{{{\mathrm{pre}}}}\right|}^{2}$ in equation (5) is replaced by the ${\left|{\mathrm{d}}{f}_{{{\mathrm{post}}}}\right|}^{2}$ in equation (6). The difference between ${\mathrm{aLFI}}_{{{\mathrm{se}}}}$ and ${\mathrm{aLFI}}_{{{\mathrm{pre}}}}$ is considered as the information gain introduced by the signal enhancement mechanism (that is, the brown bars in in Fig. 4b). Following this idea, we can calculate the stepwise aLFI by one-by-one considering the effects of manifold shrinkage ${\mathrm{aLFI}}_{{{\mathrm{ms}}}}$, signal rotation ${\mathrm{aLFI}}_{{{\mathrm{sr}}}}$ and manifold warping (${\mathrm{aLFI}}_{{{\mathrm{mw}}}}$ or ${\mathrm{aLFI}}_{{{\mathrm{post}}}}$) as

7 $${\mathrm{aLFI}}_{{{\mathrm{ms}}}}=\frac{1}{n{\Delta {\rm{\theta }}}^{2}} \frac{{\left|{\mathrm{d}}{f}_{{{\mathrm{post}}}}\right|}^{2}}{{{\underline{\lambda}}}_{{{\mathrm{post}}}}} \mathop{\sum }\limits_{i=1}^{N}\frac{{\left(\frac{{{\mathrm{d}}{f}_{{{\mathrm{pre}}}}}^T}{\left|{\mathrm{d}}{f}_{{{\mathrm{pre}}}}\right|} {\xi }_{i}^{{\,{\mathrm{pre}}}}\right)}^{2}}{{\hat{\lambda }}_{i}^{{{\mathrm{pre}}}}}$$

8 $${\mathrm{aLFI}}_{{{\mathrm{sr}}}}=\frac{1}{n{\Delta {\rm{\theta }}}^{2}} \frac{{\left|{\mathrm{d}}{f}_{{{\mathrm{post}}}}\right|}^{2}}{{{\underline{\lambda}}}_{{{\mathrm{post}}}}} \mathop{\sum }\limits_{i=1}^{N}\frac{{\left(\frac{{{\mathrm{d}}{f}_{{{\mathrm{post}}}}}^T}{\left|{\mathrm{d}}{f}_{{{\mathrm{post}}}}\right|} {\xi }_{i}^{{\,{\mathrm{pre}}}}\right)}^{2}}{{\hat{\lambda }}_{i}^{{{\mathrm{pre}}}}}$$

9 $${\mathrm{aLFI}}_{{{\mathrm{mw}}}}={\mathrm{aLFI}}_{{{\mathrm{post}}}}=\frac{1}{n{\Delta {\rm{\theta }}}^{2}} \frac{{\left|{\mathrm{d}}{f}_{{{\mathrm{post}}}}\right|}^{2}}{{{\underline{\lambda}}}_{{{\mathrm{post}}}}} \mathop{\sum }\limits_{i=1}^{N}\frac{{\left(\frac{{{\mathrm{d}}{f}_{{{\mathrm{post}}}}}^T}{\left|{\mathrm{d}}{f}_{{{\mathrm{post}}}}\right|} {\xi }_{i}^{{\,{\mathrm{post}}}}\right)}^{2}}{{\hat{\lambda }}_{i}^{{{\mathrm{post}}}}}.$$

The information gain in Fig. 4b indicates the difference between aLFI_se_, aLFI_ms_, aLFI_sr_ and aLFI_mw_ (that is, ${\mathrm{aLFI}}_{{{\mathrm{post}}}}$) as compared with the pre-test baseline ${\mathrm{aLFI}}_{{{\mathrm{pre}}}}$. They are shown as brown, blue, green and magenta bars in Fig. 4b, respectively.

### DCNN modelling of motion VPL

#### Stimuli

The experiment used random dot motion (RDM) stimuli, which consist of a cloud of independent moving dots with some degree of coherence in a given moving direction^54^. The network was trained to discriminate whether the moving direction of a target RDM stimulus was 4° clockwise or counterclockwise relative to its corresponding reference RDM stimulus. To meet the network’s specifications, the motion stimuli were 16-frame videos (112 × 112 pixels per frame). Within each frame, ~100 dots were displayed, with each dot represented by a cross of 3 pixels in both height and width. We set eight coherence levels (8.84%, 12.5%, 17.7%, 25%, 35.3%, 50%, 70.7% and 100%) and four reference directions (45°, 135°, 225° and 315°). The motion speed was 7.5 pixels per frame. All non-coherently moving dots appeared randomly in the image. The display of each frame was limited to a centred circle with a diameter of 112 pixels, with the surrounding areas displayed in black.

#### Neural network architecture and training

Our DCNN is a three-dimensional (3D) convolutional neural network inherited from the C3D network for action recognition^36^. The original C3D consists of ten convolutional layers and three fully connected layers. The main difference between C3D and AlexNet is that C3D uses 3D convolutional kernels to process spatiotemporal information. We kept the first six convolutional layers from the pretrained C3D and replaced the three fully connected layers with a fully connected layer that outputs a single scalar. The number of layers was chosen to (1) keep roughly similar number of parameters to the orientation DCNN and (2) to roughly match the number of regions of interest (ROIs) in the human neuroimaging experiment. Similar to the orientation DCNN, the motion DCNN was also configured in a Siamese fashion to perform the two-alternative forced-choice task based on the sigmoid function.

Similar to the orientation DCNN, the entire training procedure consisted of two phases: the pretraining phase and the VPL phase. During the pretraining phase, the network was trained on full-coherence noiseless RDM pairs, whereas during the VPL phase, the network was trained on stimulus pairs across all coherence levels (eight levels). The network was trained for 1,000 epochs in the pretraining phase and 2,000 epochs in the training phase using stochastic gradient descent with a learning rate of 1e^−7^, momentum of 0.9 and weight decay of 0.0005. The parameters were updated to minimize the cross-entropy loss between the network outputs and the true stimulus labels. The initial parameters in the fully connected layer were normally randomized, whereas those in the convolutional layers were taken directly from a pretrained C3D available at https://download.openmmlab.com/mmaction/recognition/c3d/c3d_sports1m_16x1x1_45e_ucf101_rgb/c3d_sports1m_16x1x1_45e_ucf101_rgb_20201021-26655025.pth. The entire procedure was repeated ten times for each reference direction to control for randomness. All model and training procedures were implemented using Python 3.10.9 conda environment, including pytorch 1.13.1, scikit-learn 1.2.0. Details of full conda environment are provided via GitHub at https://github.com/Yu-AngCheng/neural_geometry_VPL.

#### Behavioural and neural analyses

The behavioural performance of the network was also evaluated by its classification confidence (equation (1)) at all coherence levels before and after the visual training phase. In addition, the firing rates of artificial neurons were measured on each trial as the output of the ReLU layers, averaged over all locations and timepoints. All measurements were taken over 1,000 simulated trials. To ensure that units were truly driven by the stimuli, only units with a mean firing rate greater than 0.001 before and after training were included in subsequent analyses.

To perform decoding analyses, we trained a linear classifier on the firing rates of the artificial neurons to discriminate between the target and the reference stimuli. To assess the performance of the classifier, we split all trials half–half as training and test datasets, and used the average performance of the test-set. For comparison with the electrophysiological data, we calculated the Fano factor of each unit as the ratio of the variance of the firing rate to its mean, and the noise correlations as the correlation between the firing rates of units when viewing the same RDM stimulus. In addition, to measure how much information was contained in a layer per unit, we calculated the aLFI (see above).

We further validated the computational mechanisms in the motion direction discrimination task. To this end, the firing rates of the same group of units under the reference and the target stimuli were also considered as two distributions in a high-dimensional neural space. In the high-dimensional neural space, we defined signal vector, signal separation, variance, correlation, signal rotation angle, principal component (PC) strength and PC rotation angle as above.

Again, we computed linear Fisher information using a stepwise approach. For all models, we sequentially added signal enhancement, manifold shrinkage, signal rotation and manifold warping to the calculation of linear Fisher information and examined how the information within units varied with all four mechanisms. Figure 5l shows the results of the stepwise analysis in layer 6. Supplementary Fig. 3 shows the results in all six layers of the motion DCNN.

### Human fMRI experiment

The human fMRI experiment data have been published in ref. ^37^ for different research questions. The core analyses in this study beyond preprocessing and ROI definitions are specifically designed in this study. We provide relevant methods as follows and more detailed methods in Supplementary Note 4 to avoid cross-referencing.

#### Subjects and experimental procedures

A total of 22 human subjects (10 males and 12 females, ages 17–25 years) participated in the experiment. All participants had normal or correct-to-normal vision. All participants provided written informed consent, and the study obtained approval from the local ethics committee at Peking University (protocol number 2012-03-09). This study was not preregistered. All subjects were compensated 20 yuan and 100 yuan for an hour of behavioural and fMRI experiments, respectively. All participants were blinded to the study’s objectives.

All subjects were trained on a direction discrimination task (Fig. 6a; see Supplementary Note 4 for apparatus and stimulus details). The whole experiment consisted of three phases: pre-test (2 days), training (10 days) and post-test (2 days). On day 1 at pre-test and day 2 at post-test, subjects were tested on direction discrimination around 45° and 135° (angular difference 4°, 120 trials for each direction) to assess their behavioural performance before and after training. Subjects were trained on the fine-direction discrimination task for 10 days. Half of the subjects were trained at 45° and the other half at 135° (see training details in Supplementary Note 4). The assignments were randomized across subjects. Training-induced behavioural improvements have been reported in our previous work^37^. All visual stimuli were generated and presented via Psychtoolbox 3.0 in MATLAB2013A.

To assess the neural changes induced by visual training, two identical fMRI sessions were performed on day 1 at pre-test and day 2 at post-test, respectively. In each fMRI session, subjects completed four runs of the motion direction discrimination task. Each run contained 30 trials for 45° and 135° (that is, a total of 120 trials for each direction). Each run also contained 15 fixation trials, and the trial order was randomized.

#### MRI data acquisition

All MRI data were acquired using a 12-channel phase array coil on a Siemens Trio 3T scanner at Peking University. The T1-weighted anatomical data with a resolution of 1 × 1 × 1 mm³ were collected for each subject. Echo-planar imaging (EPI) functional data were collected for the motion direction discrimination task, retinotopic mapping and motion localizer experiments. EPI data were acquired using gradient echo-pulse sequences from 33 axial slices, covering the whole brain. The standard EPI sequence used for data acquisition was as follows: a repetition time of 2,000 ms, an echo time of 30 ms, a flip angle of 90° and a resolution of 3 × 3 × 3 mm³. The slice order was interleaved ascending.

In addition to the four runs of the motion direction discrimination task, we also collected one or two retinotopic mapping runs^52,55^ and a motion localizer run^55^ to define ROIs.

#### MRI data analyses

In Brain Voyager QX (version 2.8.0), the anatomical data were transformed into the Talairach coordinate space. For all functional data, the first four volumes of each functional run were discarded to allow the longitudinal magnetization to reach a steady state. The functional data underwent several standard preprocessing procedures, including slice timing correction, head motion correction, spatial smoothing, temporal high-pass filtering (generalized linear model (GLM) with Fourier basis set at two cycles) and linear trend removal. Brain Voyager QX (version 2.8.0) was also used to preprocess the data of the retinotopic mapping experiment and the motion localizer experiment. We used the standard phase-encoding method to define the retinotopic visual areas V1, V2, V3 and V3A (refs. ^56,57^). A GLM was then applied to the motion localizer data to define the motion-selective voxels (hMT+ and motion-selective voxels in IPS).

The functional data of the motion direction discrimination task were preprocessed using SPM12 (www.fil.ion.ucl.ac.uk/spm). The data were aligned to the first volume of the first run of the first session, corrected for acquisition delay and then normalized to the Montreal Neurological Institute (MNI) coordinate space using an EPI template. We used the GLMdenoise package (version 1.4, http://www.kendrickkay.net/GLMdenoise/) developed in ref. ^58^ without evoking multirun denoise procedures to estimate the single-trial activity of voxels.

#### Voxel population response analyses

We adapted the analysis previously used for artificial neurons in neural networks to the single-trial fMRI response estimates. To improve SNR, we selected the 60 most responsive voxels in each ROI at pre-test. We first investigated which ROI was involved in motion VPL by measuring the discriminability between two different motion conditions (trained direction, for example, 45° versus untrained direction, for example, 135°) before and after training. We trained a linear classifier on the fMRI data to discriminate between the two motion conditions. To assess the performance of the classifier, we performed a leave-one-trial-out cross-validation, and the average performance on the leave-out test trial was used as the discriminability measure. We also computed the average linear Fisher information (see equations above) between the 45° versus 135° conditions to quantify stimulus discriminability. We found that motion direction discrimination training significantly improved stimulus discriminability in V3A and hMT+. Therefore, we included only V3A and hMT+ voxels in the subsequently analyses.

Similar to the analyses in the DCNNs, we defined the signal vector, the signal separation, the variance, the intervoxel correlations, the signal rotation angle, the PC strength and the PC rotation angle in the multivoxel high-dimensional space using the same method defined above (Fig. 6). In addition, we applied the same stepwise analysis approach of calculating aLFI to the fMRI data (Fig. 6k).

### Monkey multiunit recording experiment

Part of the monkey psychophysical and neurophysiological data have been published in refs. ^14,59^. These previous studies showed qualitatively similar results of the learning-induced reduction in Fisher information, Fano factor and noise correlations via different analysis methods. Other results and analyses on the characteristics of population responses in this study (that is, Figs. 7 and 8), especially the validation of signal rotation and manifold warping mechanisms, as well as the stepwise information analyses, are key contribution of our study. We provide relevant methods as follows and more detailed methods in Supplementary Note 5 to avoid cross-referencing.

#### Ethics statement and data collection

The Newcastle University Animal Welfare Ethical Review Board approved all procedures in this study. All experimental procedures were carried out in accordance with the European Communities Council Directive RL 2010/63/EC, the US National Institutes of Health Guidelines for the Care and Use of Animals for Experimental Procedures and the UK Animals Scientific Procedures Act. This study included two male monkey monkeys (5 and 14 years of age). This study was not preregistered. ARRIVE guidelines were used to report the research.

#### Experimental preparation

The surgical procedure is described in ref. ^60^ and Supplementary Note 5. The headpost and electrode implementations are also described in Supplementary Note 5. In brief, in monkey 1, two 4 × 5 grids of microelectrodes were implanted in area V4; in monkey 2, one 5 × 5 grid was implanted in V4. These chronically implanted electrodes allowed us to record population activity in area V4 over the course of visual training. Importantly, we were able to record stably from a few small multiunit clusters. The stability of the recording is shown in ref. ^14^. Stable recording of multichannel neuronal activity allows analyses of changes in population responses induced by training.

#### Behavioural task and monkey training

All monkey training and data collections were conducted by CORTEX software (last updated in 2013, http://dally.nimh.nih.gov/index.html). The monkeys were trained in a contrast discrimination task in which subjects were asked to decide whether the contrast of a test stimulus was higher or lower as compared with that of a reference stimulus by making a saccade to one of two distinct locations (Fig. 7b). On each trial, the subject first kept fixation on the centre of the screen for 512 ms. After 539 ms of fixation, a vertically oriented reference Gabor stimulus with 30% contrast was presented, centred at the V4 receptive field coordinates. The outer diameter of the Gabor stimulus was truncated at 16° for monkey 1 and 14° for monkey 2. After the Gabor stimulus, monkey 2 experienced an interstimulus interval of 512 ms. By contrast, monkey 1 experienced a randomly chosen interstimulus interval, ranging from 512 to 1,024 ms. During the interstimulus interval, only the fixation dot was presented. A test stimulus was then presented for 512 ms. This test stimulus was identical in size and orientation to the reference stimulus but differed in contrast, with the contrast level chosen pseudorandomly. The test stimulus was followed by another blank period of 512 ms during which only the fixation dot was visible. After the fixation dot, two target squares, one black and one white with a size of 0.5° in size, appeared to the left and right of the location where the reference and test stimuli were previously presented. The monkeys were cued to make a decision once the fixation dot disappeared. The monkeys were required to make a saccade to the white square within a 2° × 2° window if the test stimulus had a higher contrast than the reference stimulus. Conversely, they were expected to make a saccade to the black square if the test stimulus had a lower contrast than the reference stimuli. A correct saccade was rewarded with a fluid reward, while an incorrect saccade led to no reward and a 0.2 s timeout period.

The two monkeys were first trained on an easy version (target contrast 5% or 90%) of the contrast discrimination task. After they were fully familiar with the easy task, the target contrast increased from 2 to 8, 12 and 14 levels. The data correspond to the 14 levels of target contrast (10%, 15%, 20%, 25%, 27%, 28%, 29%, 31%, 32%, 33%, 35%, 40%, 50% or 60%; Supplementary Note 5). We focus only on target contrast levels (27%, 28%, 29%, 31%, 32% and 33%) near the reference contrast (that is, 30%) according to the definition of linear Fisher information.

#### Dataset and preprocessing

We used chronically implanted Utah arrays to record spiking activity. We refer to small multiunit neuronal clusters recorded from a given electrode as channels. Twenty-nine and 20 channels were recorded in monkey 1 and monkey 2, respectively. These channels exhibited good responses (SNR >1) on over 80% of the recording sessions (see SNR computation in Supplementary Note 5). Baseline activity matching was performed between sessions for multiunit activity data to obtain comparable activity levels across sessions.

#### Behavioural and neural analyses

We noticed that the relationship between neural activity and discriminability can change drastically during the stimulus presentation period, and through training, the improvement in discriminability can also vary over the course of the training period. We chose the first four and the last four training sessions as the early and the late phase of training. This choice ensures an overall sufficient and comparable number of trials at both pre- and post-test for further analyses.

To determine the time window, we systematically varied the time window and trained a linear classifier to discriminate between the reference and target stimuli, and obtained its performance through tenfold cross-validation. We chose the time window with the largest change in decoding accuracy between the reference stimulus (30% contrast) and the target stimuli (29% or 31% contrast). For monkey 1, the chosen time window was 30–130 ms after stimulus onset. For monkey 2, the time window was 130–230 ms after stimulus onset. Note that this choice aims to maximize training effects on population representations (similar to the decoding analyses for first identifying V3A and hMT+ as the ROIs where learning effects are most pronounced in the human fMRI study) but does not guarantee the underlying mechanisms such as signal separation enhancement and manifold shrinkage. Also, varying the time window did not qualitatively change our results. We used a simple multivariate Poisson log-normal model (Supplementary Note 5, see also refs. ^61–64^) to estimate the trial-by-trial variability of population firing rates. We further use the estimated firing rates and covariance to compute all neural metrics mentioned above. We report all results in Figs. 7 and 8 for visual comparison with the DCNN and fMRI results above.

### Reporting summary

Further information on research design is available in the Nature Portfolio Reporting Summary linked to this article.

## Supplementary information

Supplementary Information Supplementary Figs. 1–7, Tables 1–7 and Notes 1–5.

Reporting Summary

## Data Availability

All data to reproduce the figures in the Article and its Supplementary Information are available via GitHub at https://github.com/Yu-AngCheng/neural_geometry_VPL. The raw human fMRI and monkey physiological data used in this study were all published previously^14,37^. Requests for other datasets should be directed to the original authors who collected the data.

## Code availability

The code for training neural networks, stimulus generation and neural geometry analysis is publicly available via GitHub at https://github.com/Yu-AngCheng/neural_geometry_VPL.
