# Supplementary material for: A neural geometry approach comprehensively explains apparently conflicting models of visual perceptual learning
Source: Nat Hum Behav. 2025 Mar 31;9(5):1023–40. doi: 10.1038/s41562-025-02149-x (PMC12106082; doi:10.1038/s41562-025-02149-x)
Supplement: Supplementary file 2 — Reporting Summary [file 41562_2025_2149_MOESM2_ESM.pdf]

nature portfolio

Corresponding author(s): Ru-Yuan Zhang

Last updated by author(s): Feb 1, 2025

Reporting Summary

Nature Portfolio wishes to improve the reproducibility of the work that we publish. This form provides structure for consistency and transparency in reporting. For further information on Nature Portfolio policies, see our Editorial Policies and the Editorial Policy Checklist.

Statistics

For all statistical analyses, confirm that the following items are present in the figure legend, table legend, main text, or Methods section.

n/a	Confirmed	
☐	☒	The exact sample size (*n*) for each experimental group/condition, given as a discrete number and unit of measurement
☐	☒	A statement on whether measurements were taken from distinct samples or whether the same sample was measured repeatedly
☐	☒	The statistical test(s) used AND whether they are one- or two-sided<br>*Only common tests should be described solely by name; describe more complex techniques in the Methods section.*
☒	☐	A description of all covariates tested
☒	☐	A description of any assumptions or corrections, such as tests of normality and adjustment for multiple comparisons
☐	☒	A full description of the statistical parameters including central tendency (e.g. means) or other basic estimates (e.g. regression coefficient) AND variation (e.g. standard deviation) or associated estimates of uncertainty (e.g. confidence intervals)
☐	☒	For null hypothesis testing, the test statistic (e.g. *F*, *t*, *r*) with confidence intervals, effect sizes, degrees of freedom and *P* value noted<br>*Give P values as exact values whenever suitable.*
☐	☒	For Bayesian analysis, information on the choice of priors and Markov chain Monte Carlo settings
☒	☐	For hierarchical and complex designs, identification of the appropriate level for tests and full reporting of outcomes
☐	☒	Estimates of effect sizes (e.g. Cohen's *d*, Pearson's *r*), indicating how they were calculated

Our web collection on statistics for biologists contains articles on many of the points above.

Software and code

Policy information about availability of computer code

Data collection	CORTEX software (last updated 2013, http://dally.nimh.nih.gov/index.html) and Psychtoolbox 3.0 in MATLAB2013A was used to collect monkey neurophysiology and human fMRI data.
Data analysis	For training deep neural networks, we used a Python 3.10.9 conda environment, including pytorch 1.13.1, scikit-learn 1.2.0. Details of full conda enviornment are provided at https://github.com/Yu-AngCheng/neural_geometry_VPL. For MRI preprocessing, we use SPM 12 and Brain Voyager QX (version 2.8.0). GLMdenoise package (version 1.4, http://www.kendrickkay.net/GLMdenoise/) was used to perform single trial general linear modeling. For monkey neurophysiology analysis, we use custom python code provided at https://github.com/Yu-AngCheng/neural_geometry_VPL.

For manuscripts utilizing custom algorithms or software that are central to the research but not yet described in published literature, software must be made available to editors and reviewers. We strongly encourage code deposition in a community repository (e.g. GitHub). See the Nature Portfolio guidelines for submitting code & software for further information.

Data

Policy information about availability of data

All manuscripts must include a data availability statement. This statement should provide the following information, where applicable:

- Accession codes, unique identifiers, or web links for publicly available datasets
- A description of any restrictions on data availability
- For clinical datasets or third party data, please ensure that the statement adheres to our policy

All data to reproduce the figures in the main and supplementary information can be found at https://github.com/Yu-AngCheng/neural_geometry_VPL. The raw fMRI data and the raw monkey neurophysiological data are available upon reasonable request, which is consistent with the original published studies.

Research involving human participants, their data, or biological material

Policy information about studies with human participants or human data. See also policy information about sex, gender (identity/presentation), and sexual orientation and race, ethnicity and racism.

Reporting on sex and gender	We collected information about the (biological) sex of the participants (self-reported), and kept the gender balance of the subjects roughly Twenty-two (12 females and 10 males) with no history of neurological or psychiatric disorders were recruited in the human fMRI experiment. We did not make any analysis of the gender of the subjects since this was beyond the scope of our research.
Reporting on race, ethnicity, or other socially relevant groupings	Participants were not classified into different race, ethnicity of other social categories.
Population characteristics	A total of 22 human subjects (10 males and 12 females, ages 17-25) participated in the experiment. All participants had normal or correct-to-normal vision. None of the participants were aware of the study's objectives.
Recruitment	We recruited participants through an online advertisement placed on a university campus bulletin board system (BBS). They were primarily undergraduate or graduate students from the universities. There was no self-selection bias.
Ethics oversight	The study obtained approval from the local ethics committee at Peking University (Potocol#: 2013-02-09).

Note that full information on the approval of the study protocol must also be provided in the manuscript.

Field-specific reporting

Please select the one below that is the best fit for your research. If you are not sure, read the appropriate sections before making your selection.

☐ Life sciences ☒ Behavioural & social sciences ☐ Ecological, evolutionary & environmental sciences

For a reference copy of the document with all sections, see nature.com/documents/nr-reporting-summary-flat.pdf

Behavioural & social sciences study design

All studies must disclose on these points even when the disclosure is negative.

Study description	This quantitative study examines whether tuning curve changes and/or noise correlation changes occur in the human brain over the course of visual training, and whether these two factors actually contribute to the improvements of population representations over the course of visual training.
Research sample	A total of 22 human subjects (10 males and 12 females, ages 17-25) participated in the experiment. All participants had normal or correct-to-normal vision. None of the participants were aware of the study's objectives. The study was previous published in Jia, K. et al. (2018). Therefore, the 22 participants are representative in terms of visual perception.
Sampling strategy	A convenience sampling is adopted, meaning the participants volunteered to attend the current study after seeing the advertisement. Sample size are chosen as comparable to previous visual perceptual learning studies using fMRI. The data were previously published in Jia, K. et al. (2018).
Data collection	The random dot motion stimuli were presented to the participants on different display devices. In the behavioral sessions, a 40 cm wide CRT monitor with a resolution of 1024 × 768 pixels and a refresh rate of 60 Hz was used. In addition, a 48 cm wide LCD projector with a resolution of 1024 × 768 pixels and a refresh rate of 60 Hz was used to display the stimuli in the fMRI sessions. In all behavioral experiments, the participants were accompanied only by the researchers. In the fMRI experiments, the participants were accompanied only by the researchers and extra MRI technicians. A single-blind design was employed, i.e., participants were unware of the purpose of this study.
Timing	2013.01-2013.11

Data exclusions	Three participants in the fMRI were excluded due to drop-out and excessive head motion which was established prior to data collection. This exclusion criteria is common in fMRI research.
Non-participation	One participant dropped.
Randomization	We only recruit one group of 22 subjects and therefore no group randomization was performed. However, trials of different conditions are randomized.

Reporting for specific materials, systems and methods

We require information from authors about some types of materials, experimental systems and methods used in many studies. Here, indicate whether each material, system or method listed is relevant to your study. If you are not sure if a list item applies to your research, read the appropriate section before selecting a response.

Materials & experimental systems

n/a	Involved in the study	
☒	☐	Antibodies
☒	☐	Eukaryotic cell lines
☒	☐	Palaeontology and archaeology
☐	☒	Animals and other organisms
☒	☐	Clinical data
☒	☐	Dual use research of concern
☒	☐	Plants

Methods

n/a	Involved in the study	
☒	☐	ChIP-seq
☒	☐	Flow cytometry
☐	☒	MRI-based neuroimaging

Animals and other research organisms

Policy information about studies involving animals; ARRIVE guidelines recommended for reporting animal research, and Sex and Gender in Research

Laboratory animals	Two male macaque monkeys (5 and 14 years of age) were used in this study.
Wild animals	The study did not involve wild animals
Reporting on sex	Sex was not considered as a factor in the current study.
Field-collected samples	The study did not contains samples collected from the field
Ethics oversight	All procedures were approved by the Newcastle University Animal Welfare Ethical Review Board and carried out in accordance with the European Communities Council Directive RL 2010/63/EC, the US National Institutes of Health Guidelines for the Care and Use of Animals for Experimental Procedures and the UK Animals Scientific Procedures Act.

Note that full information on the approval of the study protocol must also be provided in the manuscript.

Plants

Seed stocks	*Report on the source of all seed stocks or other plant material used. If applicable, state the seed stock centre and catalogue number. If plant specimens were collected from the field, describe the collection location, date and sampling procedures.*
Novel plant genotypes	*Describe the methods by which all novel plant genotypes were produced. This includes those generated by transgenic approaches, gene editing, chemical/radiation-based mutagenesis and hybridization. For transgenic lines, describe the transformation method, the number of independent lines analyzed and the generation upon which experiments were performed. For gene-edited lines, describe the editor used, the endogenous sequence targeted for editing, the targeting guide RNA sequence (if applicable) and how the editor was applied.*
Authentication	*Describe any authentication procedures for each seed stock used or novel genotype generated. Describe any experiments used to assess the effect of a mutation and, where applicable, how potential secondary effects (e.g. second site T-DNA insertions, mosiacism, off-target gene editing) were examined.*

Magnetic resonance imaging

Experimental design

Design type	Task state. Event-related design.
Design specifications	Data was first published in Jia et al. 2018, and was reanalyzed for this paper. In each fMRI session, subjects completed

Design specifications

four runs of the motion direction discrimination task. Each run contained 30 trials for 45º and 135º (i.e., a total of 120 trials for each direction). Each run also contained 15 fixation trials and the trial order was randomized. In addition to the four runs of the motion direction discrimination task, we also collected one or two retinotopic mapping runs and a motion localizer run to define regions-of-interest.

Behavioral performance measures

Subjects were tested on direction discrimination around 45º and 135º (angular difference 4º, 120 trials for each direction) to assess their accuracy.

Acquisition

Imaging type(s)

functional

Field strength

All MRI data were acquired using a 12-channel phase array coil on a Siemens Trio 3T scanner at Peking University.

Sequence & imaging parameters

The T1-weighted anatomical data with a resolution of 1 × 1 × 1 mm³ were collected for each subject. Echo-planar imaging (EPI) functional data were collected for the motion direction discrimination task, retinotopic mapping, and motion localizer experiments. EPI data were acquired using gradient echo-pulse sequences from 33 axial slices, covering the whole brain. The standard EPI sequence used for data acquisition was as follows: a repetition time of 2000 ms, an echo time of 30 ms, a flip angle of 90º, and a resolution of 3 × 3 × 3 mm³. The slice order was interleaved ascending.

Area of acquisition

Whole brain

Diffusion MRI ☐ Used ☒ Not used

Preprocessing

Preprocessing software

In Brain Voyager QX(version 2.8.0), the anatomical data were transformed into the Talairach coordinate space. For all functional data, the first four volumes of each functional run were discarded to allow the longitudinal magnetization to reach a steady state. The functional data underwent several standard preprocessing procedures, including slice timing correction, head motion correction, spatial smoothing, temporal high-pass filtering (GLM with Fourier basis set at 2 cycles), and linear trend removal. Brain Voyager QX (version 2.8.0) was also used to preprocess the data of the retinotopic mapping experiment and the motion localizer experiment. We used the standard phase-encoding method to define the retinotopic visual areas V1, V2, V3, and V3A 44,45. A generalized linear model (GLM) was then applied to the motion localizer data to define the motion-selective voxels (hMT+ and motion-selective voxels in IPS).
The functional data of the motion direction discrimination task were preprocessed using SPM12 (www.fil.ion.ucl.ac.uk/spm). The data were aligned to the first volume of the first run of the first session, corrected for acquisition delay, and then normalized to the MNI coordinate space using an EPI template. We used the GLMdenoise package developed in ref. 46 without evoking multirun denoise procedures to estimate the single trial activity of voxels.

Normalization

The data were aligned to the first volume of the first run of the first session, corrected for acquisition delay, and then normalized to the MNI coordinate space using an EPI template.

Normalization template

The functional data were normalized to the MNI coordinate space using an EPI template.

Noise and artifact removal

The functional data underwent several standard preprocessing procedures, including slice timing correction, head motion correction, spatial smoothing, temporal high-pass filtering (GLM with Fourier basis set at 2 cycles), and linear trend removal.

Volume censoring

For all functional data, the first four volumes of each functional run were discarded to allow the longitudinal magnetization to reach a steady state.

Statistical modeling & inference

Model type and settings

GLMdenoise package () was used without evoking multirun denoise procedures to estimate the single trial activity of voxels.

Effect(s) tested

Effected were test before and after visual perceptual learning

Specify type of analysis: ☐ Whole brain ☒ ROI-based ☐ Both

Anatomical location(s)

We used functional ROIs, identified and tested using retinotopic mapping runs and motion localizer runs.

Statistic type for inference

(See Eklund et al. 2016)

cluster-wise (ROI-wise) analysis were applied

Correction

We did not run whole brain search analysis. Therefore, no correction were applied since we only focused on V3a and hMT+

Models & analysis

n/a	Involved in the study
☒	☐ Functional and/or effective connectivity
☒	☐ Graph analysis
☐	☒ Multivariate modeling or predictive analysis

Multivariate modeling and predictive analysis

Single trial activity of voxels within the ROIs were used to predict the conditions of the experiment (45º and 135º). 60 voxels were used for each ROI. A leave-one-trial-out cross validation was applied. The average accuracy over the test trials was used as the metric.
